# Dependency Distance Differences across Interpreting Types: Implications for Cognitive Demand

**DOI:** 10.3389/fpsyg.2017.02132

**Published:** 2017-12-12

**Authors:** Junying Liang, Yuanyuan Fang, Qianxi Lv, Haitao Liu

**Affiliations:** ^1^Department of Linguistics, Zhejiang University, Hangzhou, China; ^2^Centre for Linguistics and Applied Linguistics, Guangdong University of Foreign Studies, Guangzhou, China; ^3^Ningbo Institute of Technology, Zhejiang University, Ningbo, China

**Keywords:** dependency distance, interpreting types, treebank, working memory, cognitive demand

## Abstract

Interpreting is generally recognized as a particularly demanding language processing task for the cognitive system. Dependency distance, the linear distance between two syntactically related words in a sentence, is an index of sentence complexity and is also able to reflect the cognitive constraints during various tasks. In the current research, we examine the difference in dependency distance among three interpreting types, namely, simultaneous interpreting, consecutive interpreting and read-out translated speech based on a treebank comprising these types of interpreting output texts with dependency annotation. Results show that different interpreting renditions yield different dependency distances, and consecutive interpreting texts entail the smallest dependency distance other than those of simultaneous interpreting and read-out translated speech, suggesting that consecutive interpreting bears heavier cognitive demands than simultaneous interpreting. The current research suggests for the first time that interpreting is an extremely demanding cognitive task that can further mediate the dependency distance of output sentences. Such findings may be due to the minimization of dependency distance under cognitive constraints.

## Introduction

Interpreting, especially simultaneous interpreting (SI), is a particularly demanding language processing task for the cognitive system underpinning language abilities. Such difficulties include the intensity and continuity of new speech input (Christoffels et al., 2006; Dong and Zhong, 2017), the general temporal overlap (simultaneity) of listening, retaining, comprehending (sometimes referred to as encoding) the input (Seeber and Kerzel, 2011), orally rendering the production, and the conflict and intervening effect of the concurrent activation of two languages (Gerver, 1976; Lambert, 1992; Padilla et al., 1995; Christoffels and De Groot, 2004; Christoffels et al., 2006; Dong and Liu, 2016). It is postulated that these cognitive underpinnings of interpreting require types of attention-sharing and overloading of working memory that people generally find very difficult (Cowan, 1995; Gile, 2008) and thus form the foci of the article in this issue (Obler, 2012).

To capture and illustrate the cognitive demands inherent to interpreting processes, several models have been proposed to conceptualize increments in the overall cognitive load, all largely synonymous with working memory. The *Process Model* (Darò and Fabbro, 1994) emphasizes the impaired sub-vocal rehearsal within working memory due to phonological interference, and the *Embedded Processes Model* (Mizuno, 2005) stresses the central executive of working memory and the long-term memory overlap with language comprehension and the production system during SI. The *Effort Model* (Gile, 2009) claims that the “memory effort,” a concept distinct from but in many ways similar to working memory, affects all facets of interpreting, including the analysis and interpretation of discourse in the input language, reformulation from the input to the target language, storage, production, and control. *The Cognitive Load Model* (Seeber, 2011; Seeber and Kerzel, 2011) measures the online memory load generated by the working memory of constituents prior to their integration and/or production through pupil dilation.

The sheer complexity of interpreting as extreme language use (Christoffels et al., 2006) also gives rise to the question in psychological domains about whether interpreters possess some special abilities that allow them to interpret successfully (Mackintosh, 1985; Christoffels et al., 2006; Russo, 2011). This question has led to empirical efforts to identify the qualities that set interpreters apart from novice or non-interpreters (Ericsson, 2000; Moser-Mercer et al., 2000; Liu et al., 2004; Christoffels et al., 2006; Cai et al., 2015). Work in this line of research consistently suggests that one possible candidate for the core component of expertise is working memory (Padilla et al., 1995; Bajo et al., 2000; Christoffels and De Groot, 2004; Köpke and Signorelli, 2011; Tzou et al., 2011).

Although prior studies have elaborated on the role of working memory in interpreting, very few studies have examined how the memory load caused by different types of interpreting affects the behavioral output of the interpreting process, in particular, the characteristics of the interpreted sentences. Exploring this question can increase understanding of how working memory affects the interpreting process. To answer this question, the present study intends to examine the characteristics of interpreted sentences across three different interpreting types, namely, *simultaneous interpreting* (SI), *consecutive interpreting* (CI), and *read-out translated speech* (TR), all based on a natural language corpus. SI is an increasingly common service for international meetings such as the General Assembly of the United Nations and other diplomatic and commercial meetings. As defined by Pöchhacker (2011a), SI is produced in synchrony with the interpreter's perception and comprehension of the original utterance, with a processing-related time lag of a few seconds between original utterance and interpretation. The simultaneity of language comprehension and production imposes a large burden on the interpreter's cognitive resources (Mizuno, 2005; Padilla et al., 2005). Different from SI, CI can be described as a two-stage process, that is, the source-speech comprehension is followed by the re-expression in another language (Gile, 2009; Pöchhacker, 2011b). This mode of interpreting is performed in such cases where speakers prefer not to “pause for interpretation” (Pöchhacker, 2011b), such as international press conferences. Faced with the need to render speeches lasting up to 20 min or more, interpreters may resort to note-taking to assist phonological memorization. While, TR is a special type of interpreting in terms of working mode: interpreters read out previously-prepared translated texts rather than interpreting impromptu. Compared with SI and CI, TR ensures the preciseness and accuracy of output texts to a large extent, and TR is normally conducted in government work reports when speakers read the speech which has also been given to interpreters in advance.

If working memory is significant for interpreting, especially in the process of generating sentences under the high constraints of cognitive resources, then it is very likely that the sentences generated under interpreting with different memory load should give rise to different characteristics of sentences. A possible scenario for a better portrayal of the cognitive outputs during interpreting is to adopt an index that varies as the processing requirements during interpreting, also with psychometric validity and reliability coupled with advanced statistical analysis. Dependency distance (DD), coined by Heringer et al. (1980) and later extended by Hudson (1995), is defined as the number of words intervening between two syntactically related words, or the difference between the two in linear position. In view of parsing models of dependency grammar, DD presents a means of measuring and calculating the memory burden imposed on language processing and reflects the dynamic cognitive load of language (Hudson, 1995; Liu et al., 2017). It establishes a syntactic relation directly between the word being processed and another word stored in working memory, with the latter decaying with time. This decay is viewed as one possible source of short-term memory breakdown (Brown, 1958; Baddeley and Hitch, 1974). Similar concepts for measuring processing difficulty have also been used by some phrase structure grammars (e.g., number of unclosed phrasal nodes). Two prominent examples are the principle of *early immediate constituents* (Hawkins, 1994, 2004) and the *Dependency Locality Theory* (Gibson, 1998, 2000).

Given the principle of Least Effort (Zipf, 1949), these different models and theories converge to suggest a universal tendency toward dependency distance minimization in natural languages—a propensity to syntactically structure sentences in such a way so as to minimize overall DD (Liu et al., 2017). This tendency is found across different languages (Liu, 2008; Futrell et al., 2015), genres (Wang and Liu, 2017), and also code-switching discourses (Wang and Liu, 2013), suggesting that it is impacted by external constraints, especially that of limited working memory. This impact has been established in a succession of empirical investigations in various languages (Gibson, 1998; Hsiao and Gibson, 2003; Grodner and Gibson, 2005). These experiments point to a law that sees processing load increasing with DD. The result of this law is thus a universal tendency for human beings to minimize DD. This tendency most certainly could also impact the interpreting processes.

The DD approach excels in its low cost and unlimited range of data, and it brings with it the introduction of new tools into the interpreting researcher's toolbox. A treebank of these three types of rendered English texts was established to test their mean dependency distances (MDDs). DD holds a considerable potential for measuring and calculating the difficulty of the interpreting process and has the potential to elucidate certain phenomena that remain unexplained in the body of literature on SI and CI. The current study endeavors to answer the following two questions:

1. Will the DD differ from each other among the TR, CI and SI, accompanied with the different cognitive demand?
2. Will the non-cognitive factors such as the treebank size and the MDD of input influence the MDD of interpreted texts?

The first question is intended to investigate the difference in DD across the three types of interpreting, while the seconds question intends to help rule out some confounding factors associated with the interpreted texts.

Given that DD mirrors constraints on human cognition (Liu, 2008; Jiang and Liu, 2015; Liu et al., 2017), it is very likely that the extreme cognitive demand of interpreting makes the DD of CI and SI shorter than that of TR. However, the difference in DD between CI and SI could entail two contrasting possibilities. The first possibility is that the DD of SI should be shorter than that of CI, since SI is generally regarded as imposing a greater cognitive load due to the simultaneity of language comprehension and production. The seconds possibility could be the opposite of the first one, for in SI, language production is highly constrained by the input, and hence the interference in syntactic structures between two languages, mainly from source language to target language, may significantly impact the MDD of the output speech. Prior research suggests that the MDD of Chinese is higher than that of English (Liu, 2008; Jiang and Liu, 2015; Liu et al., 2017), and hence it is very likely that DD of SI can be higher than CI.

## Materials and methods

**Figure 1:**
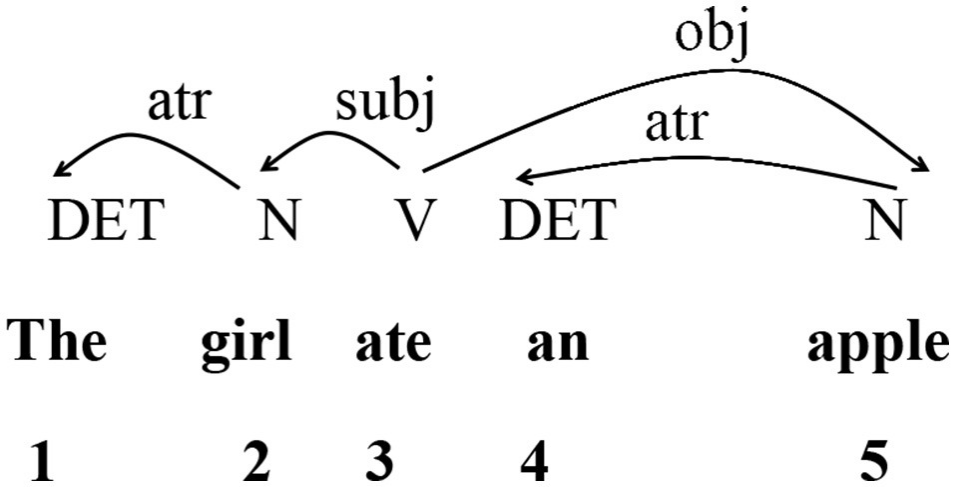
Dependency structure of sample sentence “*The girl ate an apple*.”

The present study employs DD to quantify and account for the storage cost and cognitive demands of different types of interpreting. This approach is based on the dependency relations between individual words (Tesnière, 1959; Hudson, 2007; Liu, 2009). It is generally accepted that a dependency relation has the following three core properties (Tesnière, 1959; Liu, 2009): (i) It is a binary relation between two linguistic units; (ii) It is always asymmetrical and directed, with one of the two units acting as head and the other as dependent; (iii) It is labeled, and the type of the dependency relation is usually indicated using a label on top of the arc linking the two units. Based on these three properties, a syntactic dependency tree or directed dependency graph can be constructed as the representation of the syntactic structure of a sentence. Here, we use such a directed acyclic graph to present the dependency structure of a sentence as in Figure 1. The dependency analysis for the sentence *The girl ate an apple* is illustrated below.

Figure 1 shows the dependency relations between words in a sentence. For each pair of words linked by a dependency relation, the one is called the *dependent* and the other the *governor*. The labeled arc extends from the *governor* to the *dependent* (Liu, 2008). The directed edge from *governor* to *dependent* illustrates the asymmetrical relation between these two units. The numbers below indicate the linear position of each word within the entire sentence. These numbers are used for calculating DD. Liu et al. (2009) have proposed a method for computing DD of sentences and texts. Formally, let W_1_…W_i_…W_n_ be a word string. For any dependency relation between two words, if W_a_ is a governor and W_b_ is its dependent, then the DD between them can be measured as the difference, i.e., a–b. In this way, adjacent words have a DD of 1. When “a” is greater than “b,” the DD is a positive number, indicating that the governor follows the dependent; when “a” is smaller than “b,” then the DD is a negative number and the governor precedes the dependent. But in measuring DD, the relevant measure is the absolute value of DD.

The MDD of a sentence can be defined as:

(1) $$\text{MDD}(\text{the sentence})=\frac{1}{\text{n}-1}\sum_{i=1}^{n-1}|DD_{\text{i}}|$$

Here “*n*” is the number of words in the sentence and DD_i_ means the DD of the i-th syntactic link in the sentence. In a sentence, there is generally one word in a sentence, the root verb, without a governor. The DD of this word is therefore defined as zero.

This formula can also be used to examine the MDD of a text or a treebenk:

(2) $$\text{MDD}(\text{the sample})=\frac{1}{\text{n}-\text{s}}\sum_{i=1}^{n-s}|DD_{i}|$$

In this case, *n* is the total number of words in the sample, *s* is the total number of sentences in the sample. DD_i_ is the DD of the i-th syntactic link of the text. Thus, in the sample sentence *The girl ate an apple*, a series of DDs can be obtained: 1 1 0 1 2. Each DD is obtained by subtracting the number of the word and that of its governor. Then, using Formula (1), the MDD of this sentence is obtained as 5/4 = 1.25.

Treebanks are a helpful resource for quantitatively analyzing the syntactic structures of texts and investigating how language processing is carried out (Liu et al., 2009). Hence, to examine the MDD of different types of interpreting, we built a dependency-annotated treebank of authentic speeches. The three types of interpreting texts discussed above were SI, CI, and TR. All three involved interpreting from Mandarin to English. The materials selected in our study are of similar formality in political and economic fields. Texts of SI were selected from keynote speeches presented by China's government leaders, including speeches on the UN General Debate, the Summer Davos Forum and the Boao Forum for Asia. In total, 10 speeches consisting of 32,100 word tokens comprise the SI sub-treebank. Texts of CI are from the annual press conference of two sessions (the National People's Congress and the Chinese Political Consultative Conference). During the press conference, considering the nature of the questions and answers, CI was adopted. A total of 10 texts from 2007 to 2016 are selected, with 71,327 word tokens. The sub-treebank of TR is composed of Chinese government work reports, containing 174,527 word tokens All three types of materials are from similar time span, from 2007 to 2016. An overview of the treebanks is displayed in Table 1.

**Table 1:** Overview of the Treebank.

**Interpreting type**	**Number**	**Contents**	**Size**
CI	10	The press conference of two sessions from 2007-2016	71327
SI	10	Speech on UN General Debate in 2016; Speech on Summer Davos Forum from 2012 to 2014 and in 2010 and 2016; Speech on Boao Forum for Asia from 2013 to 2016	32100
TR	10	Government work reports from 2007 to 2016	174527

These texts were transcribed and checked by graduate students majoring in English translation and interpreting to ensure accuracy and authenticity. Then these texts were entered in the Stanford Parser, a natural language parser program used to work out the syntactic structures of sentences. It describes the grammatical relationships in a sentence in a simple manner, and represents all sentence relationships uniformly as typed dependency relations. The output of grammatical relations was obtained and programmed into an EXCEL format for further computing, as presented for a sample sentence in Table 2. In Table 2, the parts of speech of words as well as the dependency relations are displayed. This kind of format facilitates the computation of DD. Hence, according to Formula (1), the MDD of the sample sentence is calculated as follows: (2 + 1 + 1 + 2 + 1 + 3) / 6 = 1.67. The materials of three types of interpreting were processed and a dependency treebank was thus built in this fashion. Manual check of the parsed result was performed before the computation of MDD. In the next section, we will present the statistical results of MDD for the three types of rendered texts.

**Table 2:** Dependency relations of sample sentence in Excel spreadsheet.

**Word Order**	**Word**	**POS**	**Word Order of Governor**	**Governor**	**POS of Governor**	**Dependency Relation**	**Dependency Distance**
1	The	DET	3	economy	NOUN	det	2
2	global	ADJ	3	economy	NOUN	amod	1
3	economy	NOUN	4	is	VERB	nsubj	1
4	is	VERB	0	is	VERB	root	−4
5	in	ADP	7	adjustment	NOUN	case	2
6	profound	ADJ	7	adjustment	NOUN	amod	1
7	adjustment	NOUN	4	is	VERB	nmod	−3
8	.	PUNCT	4	is	VERB	punct	−4

## Results

**Figure 2:**
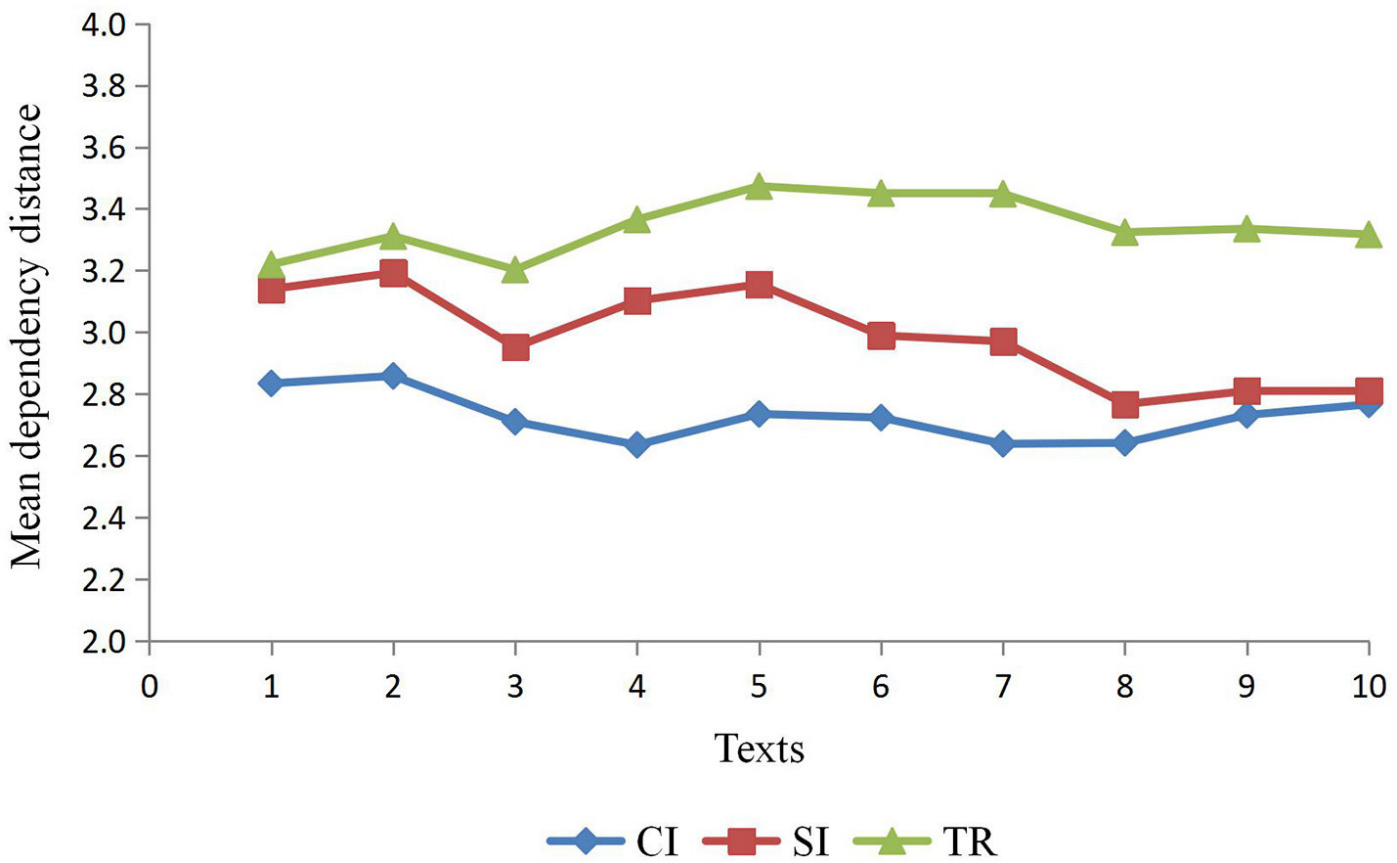
MDD for three types of outputs texts.

Table 3 lists the MDD of three types of interpreting texts, namely, CI, SI and TR, and on the basis of Table 3, Figure 2 displays a distinctive pattern of MDD for these three types of outputs texts. As is shown in Figure 2, the output texts of TR (*M* = 3.345, *SD* = 0.093) yield the highest MDD, followed by SI (*M* = 2.989, *SD* = 0.156) and CI (*M* = 2.782, *SD* = 0.078). The distribution of MDD for the three types of interpreting reveals the variance. A one-way ANOVA with MDD was performed, and it confirms the significant difference in MDD for the three types of interpreting, *F*_(2, 27)_ = 73.94, *p* < 0.001, η_*p*_^*2*^ = 0.846. Results from Tukey's *post-hoc* tests show that TR has a larger MDD than both SI (*p* < 0.001, η_*p*_^*2*^ = 0.681) and CI (*p* < 0.001, η_*p*_^*2*^ = 0.936) and SI has a larger MDD than CI (*p* < 0.001, η_*p*_^*2*^ = 0.555). It is observable from Figure 2 that though there is fluctuation in MDD, the highest MDD (3.473) is well below 4, within the threshold constrained by the working memory capacity of humans (Cowan, 2001). On the one hand, such a close approximation seems to demonstrate the constraint of human working memory during the process of interpreting, which is consistent with much prior research suggesting that dependency distance minimization could be a universal tendency in natural languages. However, on the other hand, the significant difference in MDD reveals that the memory load varies in different modes, that is to say, there exists significant difference in terms of the cognitive load among SI, CI, and TR.

**Table 3:** MDD for three types of output texts.

	**CI**	**SI**	**TR**
1	2.834	3.140	3.220
2	2.859	3.193	3.311
3	2.710	2.952	3.203
4	2.635	3.102	3.366
5	2.735	3.155	3.473
6	2.723	2.990	3.450
7	2.639	2.971	3.449
8	2.642	2.767	3.324
9	2.732	2.810	3.336
10	2.766	2.811	3.317

In our treebank, the size of texts varies from one to the other. One might argue that it is the size of the texts at hand that gives rise to the differences in MDD. To rule out the possible effect of text size on MDD, we built a new treebank with a similar size by randomly selecting from the original texts: CI texts with 33,298 words, SI texts with 32,100 words and TR texts with 32,730 words. No significant difference exists among them with regard to size, *F*_(2, 27)_ = 0.467, *p* = 0.632, η_*p*_^*2*^ = 0.033. MDD for output texts with equal sizes is computed and shown in Table 4.

**Table 4:** MDD for three types of outputs of new sizes.

	**CI**	**SI**	**TR**
1	2.834	2.952	3.373
2	2.775	2.990	3.173
3	2.675	2.971	3.322
4	2.648	3.155	3.323
5	2.701	3.102	3.535
6	2.654	3.193	3.548
7	2.615	3.140	3.418
8	2.655	2.811	3.300
9	2.683	2.767	3.464
10	2.694	2.810	3.374

**Figure 3:**
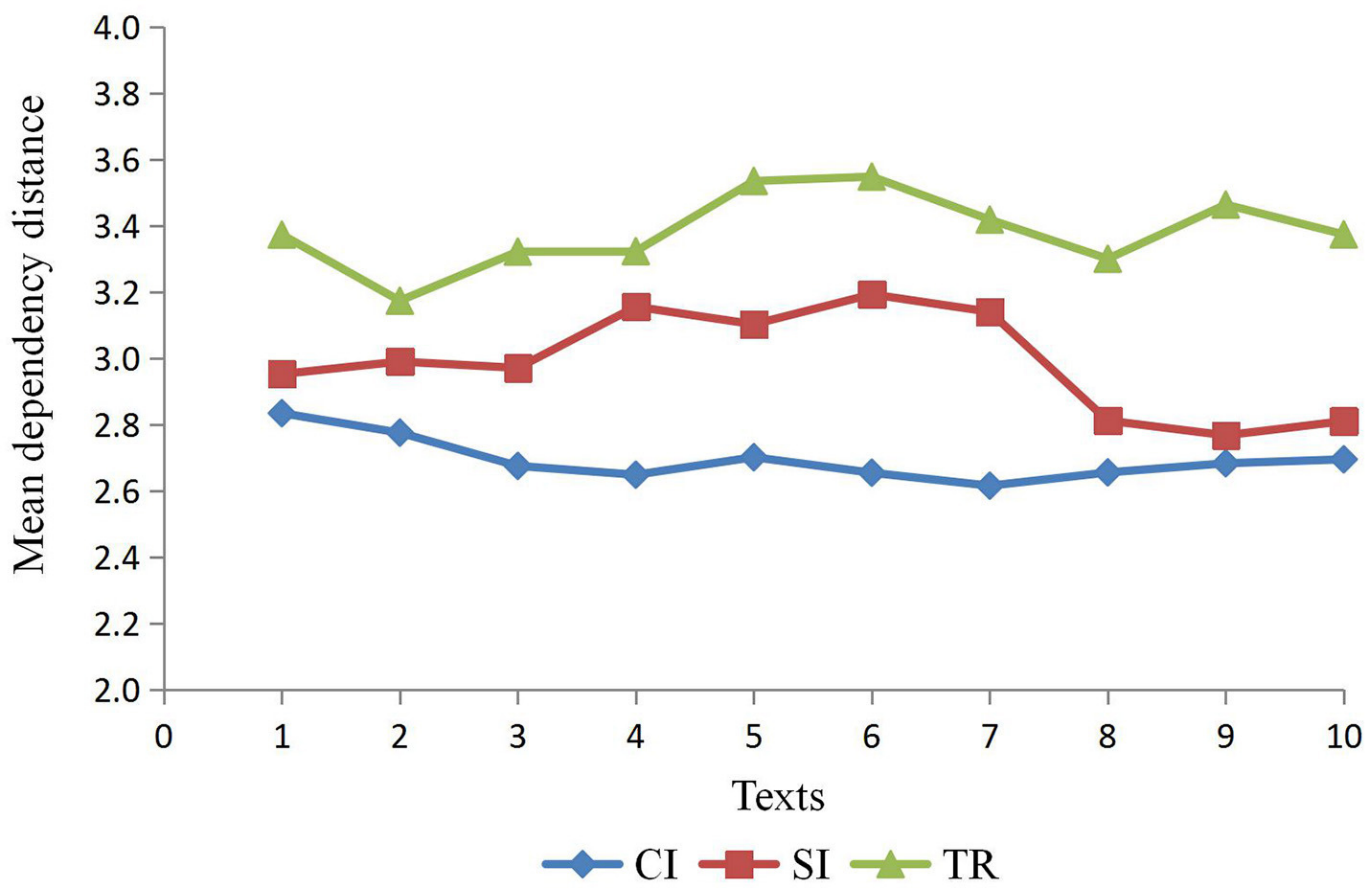
MDD for three types of output texts of new sizes.

Table 4 and Figure 3 show that MDD for three types of output texts varies from each other, in spite of the similarity in the text size. On the basis of data in Table 4, output texts of CI have the smallest MDD (*M* = 2.693, *SD* = 0.065) compared to that of SI (*M* = 2.989, *SD* = 0.156) and TR (*M* = 3.383, *SD* = 0.114). Significant differences in the MDD for three types of interpreting output still exist, *F*_(2, 27)_ = 86.431, *p* < 0.001, η_*p*_^*2*^ = 0.865. Furthermore, post-hoc tests with Tukey's correction confirm the significant difference in MDD among the three types of interpreting texts with new sizes: TR still yields a larger MDD than SI (*p* < 0.001, η_*p*_^*2*^ = 0.698) and CI (*p* < 0.001, η_*p*_^*2*^ = 0.939) and SI yields a larger MDD than CI (*p* < 0.001, η_*p*_^*2*^ = 0.629). This result is consistent with that yielded by outputs with different sizes, which indicates that the size of the texts can be ruled out as a factor that could significantly affect MDD, and it further consolidates the conclusions obtained from the original treebank in this study.

To recap, interpreting is a process mediating between source language and target language, requiring interpreters to process and produce verbal information in two languages. It might be assumed that the variance in MDD of outputs is induced by that of inputs instead of differing cognitive loads. To test this hypothesis, the MDD of corresponding input texts was calculated with the same method measuring English texts through Stanford Parser. Before further analysis of data, we first examined the parsed results.

As seen in Table 5, Chinese input texts generally compute a higher MDD than their corresponding English output texts. This result is consistent with the finding that Chinese has a higher MDD than English (Liu, 2008; Eppler, 2013; Wang and Liu, 2013; Futrell et al., 2015). Statistically, MDD of input texts in Table 5 varies in a rather limited range, *M* = 3.628 for SI, *M* = 3.583 for CI and *M* = 3.635 for TR. Furthermore, an ANOVA test confirms the non-significant difference in the MDD for the input texts, *F*_(2, 27)_ = 0.445, *p* = 0.645, η_*p*_^*2*^ = 0.032. Thus, the results here suggest that the significant difference in MDD for the output texts is induced by something other than the variances of input texts.

**Table 5:** MDD for three types of input texts.

	**CI**	**SI**	**TR**
1	3.470	3.621	3.819
2	3.579	3.828	3.497
3	3.548	3.812	3.461
4	3.587	3.552	3.511
5	3.424	3.780	3.637
6	3.598	3.492	3.598
7	3.599	3.587	3.886
8	3.605	3.415	3.746
9	3.579	3.627	3.702
10	3.839	3.569	3.489

Other factors of potential influence on the output of interpreting are the individual styles of interpreters (Besien and Meuleman, 2008) and interpreting strategies (Kajzer-Wietrzny, 2012). To examine whether individual difference contributes to the difference in MDD values, we conducted a comparison of outputs produced by different interpreters by taking the CI Treebank as an example. In our collection of CI texts, three interpreters are involved: FEI Shengchao performed the CI from 2007 to 2009, ZHANG Lu from 2010 to 2012 and from 2014 to 2016, and SUN Ning in 2013. They are all highly professional interpreters, working as commissioners of the Translation Department of China's Ministry of Foreign Affairs, and are in possession of rich experiences in political interpreting. The MDD for their outputs shows no significant difference or diversity, with 2.801 for FEI Shengchao, 2.639 for SUN Ning and 2.745 for ZHANG Lu. The result of ANOVA test rules out the possible effect of interpreting style on the MDD for output texts of the three interpreters, *F*_(2, 7)_ = 0.705, *p* = 0.526, η_*p*_^*2*^ = 0.168. Hence, the individual interpreting style of various interpreters is ruled out as a significant variance on MDD.

Overall, our statistical results show that MDD for the three types of interpreting output texts is significantly different, with TR texts yielding the largest MDD than CI and SI, and SI larger than CI. This result remains consistent in the face of factors such as the output sizes of texts, the input texts, and the interpreting style.

## Discussion

The present study examines the DD of SI, CI, and TR, based on a treebank comprising these three types of interpreting output texts with dependency annotation. Our results indicate that these three output texts entail different MDDs, with TR having the largest MDD and CI the smallest, regardless of the output sizes of texts, the input texts, or the interpreting style. This study complements previous behavioral studies in quantitatively examining the relation between cognitive load and interpreting and also suggests for the first time that interpreting, as an extremely demanding cognitive task, could further influence the DD of output sentences. Given that our minds tend to minimize the DD for created sentences due to the limited cognitive resource, the current findings can be explained by dependency distance minimization that occurs when confronted with the varying cognitive demands during interpreting.

Among the three types of interpreting in our treebank, SI has been recognized as an extremely demanding cognitive task (Christoffels and De Groot, 2005; Seeber, 2011; Macnamara and Conway, 2015; Morales et al., 2015; Gile, 2016), as many processes are performed concurrently, in different linguistic codes and under strong temporal pressure. The interpreter must attend to various tasks, including comprehension, planning, task switching, and reasoning (Mizuno, 2005; Padilla et al., 2005; Macnamara and Conway, 2015). The simultaneity of these tasks contributes to the complexity of SI (Christoffels and De Groot, 2005). On the other hand, CI requires a non-simultaneous, but sequential alternation between listening and speaking, which is the main difference between SI and CI, that is, the different timing between input and output (Christoffels and De Groot, 2005). Hence, a lesser degree of multiple task coordination is demanded in CI (Strobach et al., 2015). For TR in our treebank, since the texts have been translated prior to the speech making, the pressure on working memory disappears (Gile, 2009).

Consistent with our prediction above in the introduction, the results we obtained show that TR does entail a larger MDD than CI and SI. The risk of cognitive saturation is much lower in translation than in either mode of interpreting (Gile, 2009). Modes of input contribute to the typical difference between translation and interpreting, for the source text in translation is permanently available while the source speech for interpreting is irretrievable once missed (Christoffels and De Groot, 2005; Gile, 2009). Hence, the main reasons for the MDD of TR being highest among the three renditions are that some pressure on working memory disappears because of previous preparation on the one hand, and TR requires a higher textual density and linguistic acceptability on the other hand, such that the speech can be made with much polish and sophistication and, consequently, gives rise to a highest MDD. This finding echoes Liu et al.'s findings (2017), suggesting that when dependency distance minimization has to be sacrificed for the sake of reliable and effective communication—for instance for the sake of sophistication in translation—it may exploit other strategies and thus leads to some unique linguistic patterns with long dependencies.

However, with regard to the MDD values across SI and CI, contrary to our intuition, CI has the smallest MDD. This is consistent with the seconds possibility mentioned in the introduction above, that is, in SI, language production is highly constrained by the input, and consequently the syntactic structure of the source language may have an essential impact on that of the target language. The result of MDD difference between SI and CI is counter-intuitive, yet it is fairly within comprehension. The reason could lie in the different cognitive demand between SI and CI, and here we propose a revised effort model adapted from Gile (2016), which is illustrated next.

SIM = L + M + P + C+TC(-SR)
  L: Listening M: Short-term memory P: Production C: Coordination TC: time constraint SR: sentence reformulation

CONs =
1) Comprehension phase: L + M + NP + C+TC(-SR)
    NP: Note Production
2) Reformulation phase: NR + M + P + C+SR(-TC)
    NR: Note Reading SR: Speech Reconstruction from Memory.

In this revised model, the implicit demand from time constraints and its correlation with sentence reformation are reflected. We suggest that there is a direct correlation between time-constraint and sentence reformulation, and memory demands do exist in the reformulation phase of CI.

For SI, simultaneity and temporal pressure are two main reasons accounting for its extreme cognitive load (Padilla et al., 2005; Morales et al., 2015). The two also induce simultaneous interpreters to produce syntactic structures of the target language closely in line with those of the source language. In SI, output is highly constrained by input as interpreters handle the source speech in speech segments or chunks formulated by several words or phrases. Hence, the interference in syntactic structures between the two languages, mainly from source language to target language, has an essential impact on the MDD for the output speech. One previous study (Liu, 2008) based on dependency treebanks of 20 languages reveals that among these languages, Chinese has the highest MDD of 3.662, while English has an MDD of 2.543. The mode of SI forces interpreters to produce the output speech closely following the input speech, which may result in syntactic similarity across the source and target languages and thus influence the MDD of the output speech.

By contrast, consecutive interpreters receive speakers' uninterrupted utterances in portions of at least a few sentences, with each portion constituting a “micro” text. Interpreters in this mode process the input speech into notes for subsequent rereading, leading to temporally separated speech comprehension and speech production, which well distinguishes CI from SI. Gile points out that in the listening phase paced by the speaker, consecutive interpreters need to coordinate listening, short-term memory and note-taking efforts within time constraints, mostly coinciding with SI with the one exception of note taking. In contrast, in the reformulation phase, consecutive interpreters are described as “self-paced” (Gile, 2005), although a strong time constraint still exists as the speaker is waiting to continue the speech. However, the fact that more time and energy available for CI in the reformulation phase does not necessarily indicate less intensive processing capacity than SI. For CI, due to the manual nature of note-taking, more time is required but only part of the information can be taken down, thus generating a higher pressure on working memory (Gile, 2009). In the reformulation phase, as highlighted above, consecutive interpreters are more self-paced. Unlike the sentence-by-sentence pattern in SI, CI formulates the target speech independently, with fewer syntactic constraints from the source speech. Thus, to deal with the high working memory burden generated by the temporal constraint as well as the insufficient note-taking information, consecutive interpreters may have a strong preference for syntactic structures with a smaller DD to lessen the burden on working memory and processing difficulty. This preference is consistent with the universal preference for dependency distance minimization for human languages (Liu, 2008; Futrell et al., 2015), which is generally considered as shaped by the principle of Least Effort (Zipf, 1949). Thus, the smallest MDD generated by the rendition of CI can be considered as a combined product of avoiding potential threats of cognitive saturation as well as following the principle of Least Effort.

When it comes to cognitive saturation, we need to revisit the limited resources of cognition in interpreting (e.g., Kahneman, 1973). Basically, the mechanism of interpreting may work in the way towards cognitive proficiency, whereas unavoidable cognitive increments do exist. Here we suggest a possible “cognitive load relief” (Gile, 2008, 2009) process in SI and a cognitive load accumulation process in CI. According to Cowan (1999), working memory is a temporarily active part of long-term memory. His model puts emphasis on focus of attention with limited capacity, the mechanism which retrieves information using the cue outside of focus (Cowan, 2001). Following the assumption of Mizuno (2017), the number of chunks held in the focus of attention is deemed as a proxy of cognitive load derived from comprehension, reformulation, and production processes. Here in the present study, the possible processing model of SI and CI can be postulated respectively based on Cowan's model of working memory (Cowan, 2000, 2001). In the process of SI, the cognitive load of processing and retaining each chunk is relieved once they are interpreted. On the contrary, more chunks of information need to be kept in the focus of attention before they can be integrated into a coherent target speech sentence in CI. Thus, the total cognitive load on CI may keep accelerating and accumulating during the course. Since the processing load usually increases with the distance of dependency, the accumulating cognitive load in CI may thus also account for interpreter's preference for a smaller DD.

Furthermore, our study excludes some possible variables that may influence the MDD of the three renditions. Firstly, while we ensure the homogeneity of our texts in contents, their sizes differ from each other significantly, thus we test whether this factor would have a significant influence upon the MDD of renditions. Our results show that the MDDs for the three types of output texts remain significantly different even if their sizes are comparable. This result is in line with the finding that the mean sentence length, the absence of crossing arcs, and the grammar itself contribute together to influence the MDD of a sentence or a text (Liu, 2008; Liu et al., 2017), without mentioning the treebank size. As a matter of fact, Liu's research (Liu, 2008) used treebanks with mixed sizes; these treebanks did not show any potential effect on the conclusions. Secondly, since language comprehension and language production in interpreting and translation take place in two different languages, we hereby investigated whether the MDD of output texts is influenced by that of input texts. In our treebank, Chinese and English are two genetically different languages, with Chinese, the Sino-Tibetan family and English, the Indo-European family. Albeit that there may be a universal preference for dependency distance minimization, previous research has also verified that MDD is cross-linguistically different (Hiranuma, 1999; Liu, 2008; Futrell et al., 2015) and the MDD of Chinese is higher than that of English (Liu, 2008; Futrell et al., 2015). Based on our data, no significant variance exists among the three types of input texts, whereas significant differences exist among the three corresponding output texts, suggesting that the variance of output texts in MDD is caused by the interpreting process rather than the input texts. We further considered the probable effect of individual interpreter's style. There is a collection of evidence supporting the influence of individual differences in working memory capacity on dependency resolution processes, especially in long-distance dependency resolution (Nicenboim et al., 2015, 2016). Hence, ignoring individual differences may confound the final results. In view of individual differences in dependency resolution, we examined the MDD of renditions produced by different interpreters, and the results proved otherwise. Taken together, these results demonstrate that MDD differs across different interpreting types regardless of non-cognitive factors such as the data size or the interpreting style.

The current research is, at least to our knowledge, the first treebank-based study focusing on interpreting. In his work, Gile has observed that many answers to the questions concerning processing capacity are not available because of “the paucity of quantitative studies of processing capacity in interpreting” (Gile, 2009). Our research is an initial attempt to apply quantitative methods to exploring cognitive processes in interpreting and translation, which may complement previous qualitative studies in this field. Moreover, the application of a treebank that allows for the exploration of DD values is a main innovation in our study. As many researchers in linguistics now concur, treebanks are a useful resource for analysis of syntactic structures as well as for human language processing (Abeille, 2003; Liu et al., 2009), thus a wealth of treebank-based research has been carried out in linguistics (Ferrer-i-Cancho, 2004; Liu, 2007, 2008; Wang and Liu, 2013; Jiang and Liu, 2015; Lu and Liu, 2016), with DD as an indicator of syntactic difficulty as well as of memory burden. In light of previous studies and findings in linguistics, we have now combined this approach with research into interpreting in an effort to shed some light on interpreting studies.

## Conclusion

The current research compares MDD across three types of interpreting outputs. Our results show that, TR entails the largest MDD, suggesting that from a quantitative perspective, the risk of cognitive saturation is much lower in translation than in the other two modes of interpreting. Moreover, contrary to our expectation, CI yields the shortest MDD (instead of SI). We reason that for SI, its sentence-by-sentence mode makes it more influenced by the MDD of the input texts, whereas for CI, with its “self-paced” (Gile, 2009) mode, is less constrained by the input texts on the one hand, and on the other hand, the smallest MDD yielded by CI is in line with the principle of Least Effort (Zipf, 1949). The distinctive research method in the present study, i.e. treebank-based quantitative analysis, offers new possibilities for the quantitative analysis of interpreting. Besides, linguists and scholars in the field of interpreting and translation studies can further investigate DD in relation to working memory during interpreting. Future studies can explore further issues. For example, how do interpreters deal with sentences with high DDs? How does DD differ in other language pairs? To sum up, we need to apply this approach to more materials and across more languages, to get more solid results in terms of interpreting universals.

## Author contributions

JL and HL conceived and designed the experiments. JL and YF performed the experiments and collected the data. JL, YF, QL, and HL performed the data analyses. All authors contributed to the result interpretation and manuscript writing. All authors approved the final version of the manuscript for submission.

### Conflict of interest statement

The authors declare that the research was conducted in the absence of any commercial or financial relationships that could be construed as a potential conflict of interest.
